# “Swipe & slice”: decoding digital struggles with non-suicidal self-injuries among youngsters

**DOI:** 10.3389/fpsyt.2024.1403445

**Published:** 2024-05-13

**Authors:** Laura Orsolini, Salvatore Reina, Giulio Longo, Umberto Volpe

**Affiliations:** Unit of Clinical Psychiatry, Department of Clinical Neurosciences/DIMSC, Polytechnic University of Marche, Ancona, Italy

**Keywords:** fear of missing out, non-suicidal self-injury, social network site, problematic social media use, risky Social Media Challenges

## Abstract

**Introduction:**

Nonsuicidal-self-injury (NSSI)-related content recently emerged on social networking sites (SNS), despite its relationship with NSSI conducts is still unclear.

**Methods:**

Hence, the current population-based cross-sectional study investigated the interplay between SNS use, NSSI content engagement, risky social media challenges (RSMCs), in a sample of 404 young adults (aged 18–24), focusing on the influence of problematic social media use (PSMU) and fear of missing out (FoMO).

**Results:**

Around 51.5% of the samplewas engaged inNSSI-related contents on SNS, being mostly females (p<0.001), younger (p=0.005), transgender people and nonbinary people (p=0.030) and those who displayed higher PSMU (p<0.001) or FoMO (p=0.031). Around 66.2% of the sample currently practice NSSIs, predominantly among females (p<0.001), those using BeReal (p=0.012), actively looking for NSSIrelated contents on SNS (p<0.001) to be part of a group (p=0.0025) or learn how to practice NSSI (p=0.025). PSMU (p<0.001) and FoMO (p<0.001) emerged as significant predictors of NSSI content engagement, particularly among active seekers. NSSI conducts were significantly predicted by FoMO (p<0.001) and Snapchat (p=0.044), while negatively predicted by male sex (p<0.001), higher educational level (p=0.019) and age at which NSSI-related contents were firstly looked for (p=0.028).

**Discussion:**

These findings underline the need to implement preventivepolicies and targeted interventions to monitor NSSI-related contents on SNS, the impact of PSMU and FoMO on NSSI, particularly among youngsters.

## 1 Introduction

Non-suicidal self-injury (NSSI) is defined as any deliberate destruction of one’s own bodily tissues, enacted for non-suicidal reasons that are not sanctioned by social and/or cultural norms (1). In recent years, there has been a significant surge in NSSI-related content on the Internet (2, 3). Various forms of NSSI-based web content, including material on message boards, blogs, video-sharing websites, and especially on social networks, have been empirically examined (2). The COVID-19 pandemic has undoubtedly led to an increase in online activity, with youths uploading videos and posts on various social networks (4, 5). Recent research has delved into how this content is portrayed and speculated on its potential impact on at-risk populations, particularly youths who engage in self-harm and more likely may have direct access to such material (6–9).

Indeed, previous studies suggested that the engagement of young adults in health-risk behaviors is influenced by social motivations, such as the need to “belong to”, the desire for popularity, and the fear of missing out (FoMO), i.e. the feeling of apprehension that one is either not in the known or missing out on information, social events, life experiences and so forth (1, 8). Perceived social standing in the peer network, including popularity and a sense of belonging, also plays a significant role (10). Young adults with a weaker sense of peer belonging, a stronger need to belong to socially notorious groups, and/or a greater sense of FoMO may be at higher risk of being engaging in Risky Social Media Challenges (RSMCs), more likely due to the need to conform to peer group norms or avoid exclusion (6). Moreover, self-reported popularity and the need for notoriety and SNS-based approval are consistently linked to risky behaviors, often used to convey one’s “coolness” to peers. The primary reason for uploading content to social networking sites (SNS) seemed to be the need to ‘attract’ views and likes, serving as a measure of online popularity (1, 8). However, these motivators towards SNS usage and these gained SNS-mediated ‘social positions and roles’ should be considered in association to predict youth risk behavior on SNS and, consequently, their influence in being engaged in RSMCs (6, 11). Coherently, a previous study demonstrated that peer influence on substance use was stronger among emerging adults with high perceived popularity and a strong sense of belonging to peers (6, 11). Another study found that higher perceived popularity predicted increased substance use, but only when the need for popularity was also high (6, 11). Additionally, evidence suggests that the need for popularity, the need for belonging, and FoMO combined are able to predict social media use behaviors (6, 11). Therefore, identifying young adults at a higher risk of participating in RSMCs requires examining classes of individuals who share common social motivations and positions in the peer context (6, 11, 12).

Considering NSSI as a highly stigmatized and often misunderstood behavior, communication on the topic is more likely to occur in a virtual environment, where the Internet facilitates personal connections and anonymous disclosure of topics that would otherwise be difficult to discuss with peers but also family members. While online communication of NSSI may have some benefits on preventing these behaviors, on the other hand, certain contents and forms of communication on the Internet, such as NSSI/suicidality-based challenges on SNS (e.g., the Blue Whale Challenge, and others), may indeed contribute to the reinforcement of NSSI (2, 6) and determine serious consequences (13, 14).

Basic social media challenges involve recording and uploading videos of oneself performing specific behaviors and then nominating others to do the same. While some challenges have positive intentions and are relatively safe, such as the ALS ice bucket challenge, many others involve serious (and potentially fatal) risky health behaviors, such as the Cinnamon Challenge (ingesting a spoonful of cinnamon without liquid), the Tide Pod Challenge (ingesting a Tide Pod containing chemicals), and the Kiki Challenge (dancing next to a moving vehicle). Videos of youths engaging in risky social media challenges (RSMCs) have garnered millions of views on social networking sites (SNSs) like YouTube and have resulted in serious health consequences, including aspiration, poisoning, car accidents, and even death (6).

Therefore, our study aims at investigating the NSSI phenomenon mediated by SNSs by exploring the impact of a PSMU and FoMO on NSSI-related contents on SNS and/or the occurrence of NSSI behaviors, within a sample of young adults (aged 18–24) from the general population, within the SWATCH (Social Withdrawal And TeCno-mediated mental Health issues) study, aiming at assessing all web-based psychopathological mental health issues. The NSSI phenomenon was explored specifically collecting information on motivators, NSSI functions, NSSI frequency, and SNS-related NSSI contents.

## 2 Materials and methods

### 2.1 Study design and recruitment strategies

This Italian population-based observational cross-sectional study was conducted from July 2023 to October 2023. The population sample consisted of young adults (aged 18–24), according to the World Health Organization (WHO)’s definition (15). recruited from the general population by using a snowball sampling recruitment strategy. No exclusion criteria have been identified for this study, despite we originally included a question by asking all participants if they had previous contacts with mental health professionals for any reason. However, being this variable not mandatory to be filled out, we did not receive a significant number of replies by participants. Participation was anonymous and voluntary without monetary or other incentives. All participants gave informed consent to take part in the study. Sample size was calculated using the Statistical Software G*Power version 3.1. (Franz, Universitat Kiel, Germany), by keeping the values of confidence level as 99%, anticipated population proportion 0.5, an α error of 0.05, a power of 80%, and taking into consideration all variables to be entered in the multivariable analysis, in order to obtain at least an effect size of >0.6. A total sample size of 369 was established to be reached for the present study. Participants were requested to fill out a set of self-report questionnaires administered through the EUSurvey platform (https://ec.europa.eu/eusurvey/home/welcome). The total sample included 404 participants, 31 of them were excluded due to denial of informed consent and/or refusal to participate in the study. The study was conducted in accordance with the ethical principles outlined in the Declaration of Helsinki and according to the guidelines for Good Clinical Practice (GCP) (16), following the approval by the local Institutional Review Board. All participants gave informed consent to take part in the study.

### 2.2 Measurements

We collected a set of socio-demographic and clinical variables, including participants’ age, sex, gender identity and level of education (in years), use of social networks (specifically), use of SNSs and which one specifically. We also asked participants if they ever searched for Risky Social Media Challenges (RSMCs) on social networks and the main reason/motivation for looking for them. Moreover, a set of self-rated questionnaires were requested to be filled out (as described below).

The Italian Version of Bergen Social Media Addiction Scale (BSMAS) (17) is a 6-items scale used to assess problematic social media use, based on the core components of addiction (i.e., salience, mood modification, tolerance, withdrawal, conflict, and relapse) (18). The scale is rated on a 5-point Likert scale ranging from 1 “very rarely” to 5 “very often”. The scale is referred to experiences related to social media use within a time frame of 12 months. The highest is the BSMAS total score, the highest is the propensity to develop problematic social media use. We adopted a cutoff of 19 to discriminate between problematic versus non problematic use of social media (PSMU versus notPSMU), according to the study by 19. In our study, BSMAS displays a good internal reliability (Cronbach’s α= 0.761).

The Italian Version of the Fear of Missing Out Scale (FoMOs) (20) is a 10-items questionnaire on a 5-point Likert-type scale (from 1 = “not at all true of me” to 5 = “extremely true of me”), assessing individuals’ levels of FoMO on the developments they perceive on social media. The total score ranges from 10 to 50, with higher scores indicating a greater fear of missing out. A good Cronbach’s α internal consistency was described (21). In our study, FoMOs displayed a good internal reliability (Cronbach’s α = 0.794).

The Italian translation of the Inventory of Statements About Self-Injury (ISAS) (22) was used to assess the relationship between NSSI, social media use and frequency. The scale was designed to assess each NSSI function documented in the research literature comprehensively. The ISAS comprises an initial section assessing lifetime frequency of 12 NSSI behaviors performed “intentionally (i.e., on purpose) and without suicidal intent”. The behaviors assessed are banging/hitting self, biting, burning, carving, cutting, wound picking, needle-sticking, pinching, hair pulling, rubbing skin against rough surfaces, severe scratching, and swallowing chemicals (22). Participants were asked to estimate the number of times they have performed each behavior (from “never in life” to “everyday”). Those endorsing one or more NSSI behaviors were asked to assess 13 potential functions of NSSI according to the ISAS section of the questionnaire: a) affect-regulation; b) anti-dissociation; c) anti-suicide; d) autonomy; e) interpersonal boundaries; f) interpersonal influence; g) marking distress; h) peer bonding; i) revenge; l) self-care; m) self-punishment; n) sensation seeking; and, o) toughness. Each function is assessed by 3 items (from “0 = not relevant”, “1 = somewhat relevant”, and “2 = very relevant”) depending on the individual’s “experience of (non-suicidal) self-harm behavior”. The score for each of the 13 ISAS functions can range from 0 to 6. In our study, ISAS displays a good internal reliability (Cronbach’s α = 0.854).

### 2.3 Statistical analysis

Data analysis was performed using the Statistical Package for Social Science for MacOS (SPSS) Software, version 27.0 (IBM Corp., Armonk NY). All the analyses were two-sided with α of 0.05. Descriptive statistics were performed in order to describe the socio-demographic and clinical characteristics of the sample, by summarizing categorical variables as frequency (N) and percentage (%). After analyzing the continuous variables for skewness, kurtosis, normality distribution through the Shapiro-Wilk test, and the equality of variances by Levene test, parametric or non-parametric statistical tests were used, when appropriate. Normally distributed continuous variables were represented using the average mean and standard deviation (SD), whether normally distributed, or the median and 95% Confidence Interval (95% CI) when not normally distributed. To compare all socio-demographic and categorical variables in each group, the χ2 Test was used. The Analysis of Variance (ANOVA) was performed to compare both BSMAS and FoMOs total scores across all socio-demographic and categorical variables (including those derived by the ISAS questionnaire). Before specifically assessing the predictors of NSSI behavior related to SNS, two preliminary multivariate linear regression analyses were run to investigate whether socio-demographic and other SNS- and/or NSSI-related predictors are potentially associated with the probability to develop a PSMU (as assessed by using the BSMAS total score as dependent variable) and FoMO (as assessed by using the FoMOs total score as dependent variable) within our sample of young adults. Then, in order to identify potential risky and protective socio-demographic and SNS- and/or NSSI related to SNS-related factors associated with the probability to develop NSSI conducts, a stepwise binary logistic regression analysis was run within all our sample. The odds ratios (OR), corresponding to 95% of confidence intervals (CI), standardized coefficient β values were generated for each variable.

## 3 Results

### 3.1 Socio-demographic and clinical characteristics of the sample

The majority of the sample (84.7%; n = 316) were women, with around 70% (n = 261) of them who declared to be cisgender. 78.8% (n=294) reported being students. The mean age of the sample is 21.1 (SD = 2.7) and the average educational level was 13.3 (SD = 2.5). Overall, 99.7% (n = 372) of participants declared to have used at least one social network in their daily life. Snapchat (96.8%; n = 361) and Instagram (92.5%; n = 345) seemed to be the most used social media as declared by participants. The mean age of subjects who use TikTok (p < 0.001) and Snapchat (p = 0.028) is significantly lower than those who do not use them. The mean age of subjects who use Facebook (p < 0.001) and Instagram (p = 0.005) is significantly higher than those who do not use them. There is no difference in the age of participants who use Twitter (p = 0.739) or BeReal (p = 0.136) and those who do not use them. Around 92.5% (n = 345) of participants declared to know about the Blue Whale Challenge and more than half of the sample (51.5%; n = 192) referred to have looked for NSSI contents on SNS. Women more frequently reported having searched NSSI contents on SNS (p < 0.001), compared to the male counterpart. It was also observed that those searching for NSSI content less significantly use Facebook (p = 0.002), while they much more declared to use TikTok (p = 0.009) or other SNS among those not listed in the questionnaire (p = 0.01). Subjects who reported to have looked for NSSI contents on SNS are significantly younger than those who do not have searched for such contents (p = 0.005). Most of the subjects who have seen NSSI contents on SNS have sought such contents for curiosity (50.5%; n = 107) or for help/support (30.1%; n = 66). The average educational level of subjects who have seen NSSI contents on SNS for seeking help is significantly higher than those who do it for other reasons (p = 0.02). Only 4.8% (n = 18) of participants reported to actively follow accounts and/or online pages/groups posting NSSI-related contents. The average age at which participants declared they first searched for or watched NSSI-related content is 14.0 (SD = 2.0). The mean age of transgender people and non-binary subjects (p = 0.030) and females (p < 0.001) who first searched for or watched NSSI-related contents was significantly lower than cisgender subjects and males. At the same time, the average age of subjects who first searched for or watched NSSI-related contents with the purpose of being part of a group (p = 0.045) or the purpose to learn how to do NSSI (p = 0.001) is significantly lower than those who do it for other reasons. **Table 1** summarizes all the socio-demographic and clinical characteristics of the sample.

**Table 1:** Socio-demographic and clinical characteristics of the sample.

		Count	%
**Sex**	males	57	15.3%
	females	316	84.7%
**Gender**	cisgender people	261	70%
	transgender people	10	2.7%
	non binary people	102	27.3%
**Student**	no	79	21.2%
	yes	294	78.8%
**Using Social Networks**	no	1	0.3%
	yes	372	99.7%
**Use Facebook**	no	299	80.2%
	yes	74	19.8%
**Use Instagram**	no	28	7.5%
	yes	345	92.5%
**Use TikTok**	no	180	48.3%
	yes	193	51.7%
**Use Twitter**	no	344	92.2%
	yes	29	7.8%
**Use BeReal**	no	309	82.8%
	yes	64	17.2%
**Use Snapchat**	no	361	96.8%
	yes	12	3.2%
**Use Other Social Media**	no	325	87.1%
	yes	12	3.2%
**Search NSSI content**	no	181	48.5%
	yes	192	51.5%
**Search NSSI content for Curiosity**	no	95	49.7%
	yes	96	50.3%
**Search NSSI content for Seeking Help**	no	133	69.6%
	yes	58	30.4%
**Search NSSI content for Pleasure**	no	175	91.6%
	yes	16	8.4%
**Search NSSI content for Need to belong to a group**	no	162	84.8%
	yes	29	15.2%
**Search NSSI content to Learn how to do it**	no	162	84.8%
	yes	29	15.2%
**Search NSSI content for popularity**	no	184	96.3%
	yes	7	3.7%
**Search NSSI content for Other reasons**	no	167	87.4%
	yes	24	12.6%
**Follow NSSI inherent pages**	no	355	95.2%
	yes	18	4.8%
**Know Blue Whale challenge**	no	28	7.5%
	yes	345	92.5%

NSSI: Non-Suicidal Self-Injury.

### 3.2 Psychopathological characteristics of the sample

The mean score at BSMAS is 15.8 (SD = 2.7), being males who scored significantly lower at BSMAS total score (p = 0.021) compared to females. After stratifying the sample in two groups (PSMU versus notPSMU), according to the BSMAS cut-off, around 53.4% (N = 199) of participants displayed significant PSMU, being mainly females (p = 0.033). Subjects who have watched NSSI-related content on SNS scored significantly higher at the BSMAS total score than subjects who did not (p = 0.007). Participants who searched NSSI-related contents to learn how to do NSSI scored significantly higher at the BSMAS total score than subjects who did it for other reasons (p < 0.001) (**Table 2**).

**Table 2:** Socio-demographic and clinical characteristics of the sample stratified according to the BSMAS.

		M	SD	p-value
**Sex**	males	14.6	4.2	**0.021**
	females	16.0	4.3	
**Gender**	cisgender people	15.8	4.2	0.935
	transgender people	15.4	4.1	
	non binary people	15.7	4.4	
**Student**	no	15.5	3.9	0.582
	yes	15.8	4.4	
**Use Facebook**	no	15.7	4.1	0.858
	yes	15.9	4.8	
**Use Instagram**	no	14.6	4.3	0.136
	yes	15.9	4.3	
**Use TikTok**	no	15.3	4.5	0.063
	yes	16.2	4.0	
**Use Twitter**	no	15.7	4.3	0.689
	yes	16.1	4.0	
**Use BeReal**	no	15.7	4.3	0.674
	yes	16.0	4.3	
**Use Snapchat**	no	15.8	4.2	0.443
	yes	14.8	5.3	
**Use of Other Social Media**	no	15.8	4.3	0.781
	yes	15.6	4.4	
**Search NSSI content**	no	15.2	4.2	**0.007**
	yes	16.3	4.2	
**Search NSSI content for curiosity**	no	16.1	4.3	0.520
	yes	16.5	4.2	
**Search NSSI content for seeking help**	no	16.0	4.2	0.149
	yes	17.0	4.3	
**Search NSSI content for pleasure**	no	16.2	4.2	0.195
	yes	17.6	4.0	
**Search NSSI content for need to belong to a group**	no	16.1	4.3	0.063
	yes	17.7	3.6	
**Search NSSI content to learn how to do it**	no	15.9	4.2	**<0.001**
	yes	18.7	3.4	
**Search NSSI content for popularity**	no	16.3	4.2	0.842
	yes	16.0	4.0	
**Search NSSI content for Other reasons**	no	16.4	4.2	0.518
	yes	15.8	4.3	
**Follow NSSI inherent pages**	no	15.7	4.2	0.189
	yes	17.1	5.1	
**Knowledge about Blue Whale challenge**	no	16.0	4.8	0.762
	yes	15.7	4.2	

In bold significant p-values. NSSI, Non-Suicidal Self-Injury; BMAS, Bergen Social Media Addiction Scale. M, mean; SD, Standard deviation.

The mean score at FoMOs is 28.8 (SD = 2.7), without any sex-based differences (p = 0.380). Subjects who use TikTok (p = 0.002) or BeReal (p = 0.002) scored significantly higher scores at the FoMOs than subjects who declared to not use them. Subjects who have watched NSSI-related content on SNS scored significantly higher at the FoMOs total score than subjects who did not (p = 0.031) (**Table 3**).

**Table 3:** Socio-demographic and clinical characteristics of the sample stratified according to the FoMO.

		M	SD	p-value
**Sex**	males	29.5	7.4	0.380
	females	28.6	7.3	
**Gender**	cisgender people	29.1	7.0	0.378
	transgender people	28.7	10.0	
	non binary people	27.9	7.8	
**Student**	no	28.3	7.2	0.501
	yes	28.9	7.4	
**Use Facebook**	no	28.8	7.1	0.857
	yes	28.6	8.2	
**Use Instagram**	no	26.9	7.3	0.161
	yes	28.9	7.3	
**Use TikTok**	no	27.6	7.1	**0.002**
	yes	29.9	7.4	
**Use Twitter**	no	28.7	7.3	0.329
	yes	30.0	7.8	
**Use BeReal**	no	28.2	7.3	**0.002**
	yes	31.4	7.0	
**Use Snapchat**	no	28.7	7.3	0.426
	yes	30.4	6.9	
**Use Other Social Media**	no	28.7	7.3	0.873
	yes	28.9	7.5	
**Search NSSI content**	no	27.9	6.8	**0.031**
	yes	29.6	7.7	
**Search NSSI content for Curiosity**	no	30.1	8.1	0.313
	yes	29.0	7.3	
**Search NSSI content for Seeking Help**	no	29.1	7.8	0.200
	yes	30.6	7.4	
**Search NSSI content for Pleasure**	no	29.7	7.8	0.424
	yes	28.1	6.3	
**Search NSSI content for Need to belong to a group**	no	29.2	7.7	0.162
	yes	31.4	7.3	
**Search NSSI content to Learn how to do it**	no	29.1	7.7	0.054
	yes	32.1	7.1	
**Search NSSI content for popularity**	no	29.6	7.7	0.557
	yes	27.9	7.5	
**Search NSSI content for Other reasons**	no	29.8	7.8	0.246
	yes	27.8	6.9	
**Follow NSSI inherent pages**	no	28.6	7.3	0.078
	yes	31.7	7.4	
**Know Blue Whale challenge**	no	30.5	8.8	0.289
	yes	28.6	7.2	

In bold significant p-values. NSSI, Non-Suicidal Self-Injury; FoMOs, Fear Of Missing Out - Scale.M, mean; SD, Standard deviation.

According to the ISAS, 85% (n = 317) of the sample reported a history of self-injurious gestures, while 66.2% (n = 247) currently practice NSSIs. Most of the sample who currently practice NSSI declared to do it to vent (64.8%; n = 160) or to self-punishment (42.5%; n = 105). The most frequently reported methods to act NSSI were (in order of frequency): hindering wound healing (64.4%; n = 159), biting themselves (42.1%; n = 104), pinching themselves (40.1%; n = 99), scratching themselves (34.4%; n = 85) and getting cuts (31.6%; n = 78). Subjects who declared to not practice NSSIs were significantly older (p = 0.002), with higher educational level (p = 0.005) and they were older when they first searched NSSI-related contents on SNS (p < 0.001). Participants who manifested NSSI conduct scored significantly higher at the BMAS (p < 0.001) and FoMOs (p < 0.001) total scores. At the same time, subjects who currently practice NSSI declared to use much more likely BeReal (p = 0.012) and less frequently Facebook (p = 0.055), they were mostly females (p < 0.001), search more for NSSI-related contents on SNS (p < 0.001), and declared to look for NSSI-related contents on SNS more likely to be part of a group (p = 0.0025) and to learn how to practice NSSI (p = 0.025) and are mostly constituted by subjects with a PSMU (p < 0.001). Participants who currently practice NSSI to punish themselves (p = 0.011) or subjects who bite themselves (p = 0.022) or tear their hair out (p = 0.025) scored significantly higher at the BMAS total score, compared to their counterparts. Only subjects who perform NSSI by hitting themselves displayed a significantly lower educational level (p < 0.001) (**Table 4**).

**Table 4:** Clinical characteristics of the sample regarding self-harm conducts.

		Count	%
**Committed Self-injurious gestures in life**	no	56	15.0%
	yes	317	85.0%
**Practice actual self-injurious gestures**	no	126	33.8%
	yes	247	66.2%
**Self-harm to calm down**	no	218	58.4%
	yes	155	41.6%
**Self-harm to punish myself**	no	261	70.0%
	yes	112	30.0%
**Self-harm to ask for help**	no	314	84.2%
	yes	59	15.8%
**Self-harm to feel like I’m a part of a group**	no	367	98.4%
	yes	6	1.6%
**Self-harm to be popular**	no	372	99.7%
	yes	1	0.3%
**Self-harm to vent**	no	180	48.3%
	yes	193	51.7%
**Cutting**	no	295	79.1%
	yes	78	20.9%
**Scratching**	no	288	77.2%
	yes	85	22.8%
**Burn yourself**	no	347	93.0%
	yes	26	7.0%
**Bite yourself**	no	269	72.1%
	yes	104	27.9%
**Hit yourself**	no	308	82.6%
	yes	65	17.4%
**Hinder the healing of a wound**	no	214	57.4%
	yes	159	42.6%
**rubbing on the rough**	no	326	87.4%
	yes	47	12.6%
**Pinches**	no	274	73.5%
	yes	99	26.5%
**Tear your hair**	no	327	87.7%
	yes	46	12.3%
**Needles**	no	336	90.1%
	yes	37	9.9%
**Ingest harmful substances**	no	345	92.5%
	yes	28	7.5%
**Other ways**	no	318	85.3%
	yes	55	14.7%

According to the multivariate linear regression model, PSMU (as assessed by using BSMAS total score) was positively predicted by total score of the FoMOs (B = 0.204, p < 0.001) and from researching NSSI contents with the purpose of learning how to practice NSSI (B = 2.225, p = 0.005) (R = 0.441, R2 = 0.195, F(2,185) = 22.359, p < 0.001) (**Table 5**). According to another multivariate linear regression model, we found that FoMO (as assessed by using the FoMOs total score) was positively predicted by BMAS total score (B=0.695, p<0.001) and from using BeReal as predominant preferred SNS (B = 2.657, p = 0.037) (R = 0.423, R2 = 0.179, F(2,185) = 20.101, p < 0.001) (**Table 6**).

**Table 5:** Multivariate Linear Regression with BSMAS total score (as dependent variable).

	B	SE	Beta	t	p-value
**FoMOs total score**	0.204	0.036	0.373	5.596	**<0.001**
**Search NSSI contents on SNS to learn how to practice NSSI**	2.225	0.776	0.191	2.866	**0.005**

SE, Standard Error; FoMOs, Fear of Missing Out - Scale; NSSI, Non-Suicidal Self-Injury; SNS, Social Networking Site.In bold significant p-values.

**Table 6:** Multivariate Linear Regression with FoMOs total score (as dependent variable).

	B	SE	Beta	t	p-value
**BMAS total score**	0.695	0.123	0.380	5.653	**<0.001**
**Using BeReal**	2.657	1.265	0.141	2.101	**0.037**

SE, Standard Error; BMAS, Bergen Social Media Addiction Scale.In bold significant p-values.

A logistic regression analysis was performed to ascertain the effects of FoMO, PSMU and socio-demographic and clinical characteristics on the likelihood of developing NSSI conducts. The logistic regression model was statistically significant, *χ*2(2) = 54.503, p < 0.001. The model explained 40.8% (Nagelkerke R2) of the variance in subjects who commit NSSI and correctly classified 86.2% of cases. According to the logistic regression model, NSSI conducts were significantly predicted by using Snapchat (Exp(B) = 7.783; 95%IC = 1.061 - 57.09; p = 0.044) and by higher FoMO levels (Exp(B) = 1.162; 95%IC = 1.088 - 1.240; p < 0.001) and negatively predicted by male sex (Exp(B) = 0.065; 95%IC = 0.016 - 0.261; p < 0.001), higher educational level (Exp(B) = 0.778; 95%IC = 0.631 - 0.959; p = 0.019) and age at which NSSI-related contents were researched/looked for (Exp(B) = 0.770; 95%IC = 0.610 - 0.971; p = 0.028) (**Table 7**).

**Table 7:** Binary logistic regression analysis predicting NSSI conducts.

	B	SE	Wald	df	p-value	Exp (B)	95% C.I. Exp (B)	
**FoMOs total score**	0.150	0.033	20.158	1	**<0.001**	1.162	1.088	1.240
**Sex (Male)**	-2.739	0.712	14.784	1	**<0.001**	0.065	0.016	0.261
**Educational level**	-0.252	0.107	5.525	1	**0.019**	0.778	0.631	0.959
**Using Snapchat**	2.052	1.017	4.074	1	**0.044**	7.783	1.061	57.090
**Age at which NSSI content was first researched/watched on SNS**	-0.261	0.119	4.859	1	**0.028**	0.770	0.610	0.971

SE, Standard Error; df, degree of freedom; C.I., Confidence Interval; FoMOs, Fear of Missing Out - Scale; NSSI, Non-Suicidal Self-Injury; SNS, Social Networking Site.

In bold values means significant p-values.

## 4 Discussion

To the best of our knowledge, our study provides significant insights into the relationship between social media use, engagement with NSSI-related content, and the prevalence of RSMCs among young adults. In particular, our findings clearly supported our research hypothesis about a potential role of a PSMU and/or FoMO as potential risk factors in increasing the chance to be engaged with NSSI-related contents and behaviors due to SNS use, particularly in those more vulnerable people. In fact, according to our findings, it seems that being attracted by looking for NSSI-related contents on SNS also increases the likelihood to develop a PSMU due to the need to have access to this type of information. Moreover, BeReal appeared to be the most predominant SNS platform able to elicit RSMCs, at least in our study.

One key-point observation from the study is the high prevalence of NSSI-related behaviors among the surveyed sample (which indeed comes from the general population and not a clinical sample), with a substantial percentage of participants who declared a current regular engagement in NSSIs (around two third of the sample) or a previous history of NSSIs (around 85% of the sample). These results highlight the urgent need for targeted interventions and support mechanisms to address the underlying psychological distress and maladaptive coping strategies prevalent among our current youth generation (4). These findings are indeed consistent with previous research carried out during the post-COVID-19 era which demonstrated a 2-/3-fold increase in NSSI, particularly among youngsters (23–25). Among the main motivations to practice NSSIs declared by our sample, are listed the need ‘to vent’ or the ‘self-punishment’. While as the most frequently reported NSSI means were reported (in order of frequency): ‘hindering wound healing’, biting, pinching, scratching themselves or getting cuts. Our findings reported that the female sex and having a lower education level seemed to represent the main socio-demographic risk factors for manifesting NSSIs in our sample of Italian young adults (8, 19).

Furthermore, in our study, almost all participants declared to fully know and to be aware about the ‘Blue Whale Challenge’ and other RSMCs, while more than half of the sample (mainly females, younger and with a higher educational level) declared to have looked for NSSI-related contents on SNS (particularly on TikTok), mostly for curiosity or for seek help/support (7, 8). Dramatically, the mean age declared by participants on their first use of SNS to look for NSSI-related content ranges from 12 to 16-years-old, being the lower age mainly represented by trans-gender and non-binary subjects and females, in line with previous published literature (24, 26–28). The main motivations for looking for NSSI-related contents appeared to be, according to our findings, mostly age-related, i.e. the lowest is the age of participant, the highest is the probability that the participant’s declared motivation for looking for NSSI-related on SNS content is represented by the need to belong to a SNS-based peer group or the need to access to information on how to perform NSSIs (29, 30). However, only a minority of the sample (around 5%) declared to actively and regularly follow accounts posting NSSI-related contents on SNS.

Furthermore, the study found that both problematic social media use (PSMU) and fear of missing out (FoMO) seemed to act as significant predictors of youths’ engagement on NSSI-related content on SNS and behaviors. Regarding the relationship between SNS and NSSI, in our study, we found that participants who declared a current NSSI conduct significantly displayed higher risk for the development of PSMU and FoMO. These findings appeared to be in line with previous published studies (31–33). Participants who declared to have looked for and/or who currently regularly look for NSSI-related contents on SNS are those subjects who displayed significantly higher BSMAS total scores and FoMOs total scores. The highest BSMAS total scores in this sub-sample are displayed by females and among those participants who declared to actively look for NSSI-related contents on SNS with the intention to access more information on NSSI and to learn means and methods to practice NSSI in real life. These findings potentially support the research hypothesis that PSMU and/or FoMO could exacerbate individuals’ vulnerability to manifest NSSI conducts, among those who actively seek out NSSI-related contents on social media platforms (34, 35). These findings emphasize the role of social media in facilitating access to harmful content and shaping individuals’ perceptions and behaviors, as previously already documented in more vulnerable people (36).

The association between specific social media platforms and engagement with NSSI content is also noteworthy. In fact, the logistic regression model clearly evidenced that NSSI conducts are significantly predicted by using SnapChat (OR=7.8) and having a FoMO (OR=1.2). Snapchat emerges as a significant predictor of NSSI behaviors, highlighting the need for targeted interventions on platforms popular among young adults. One could argue that Snapchat could probably emerge as a possible predictor for NSSIs because those subjects who use it are those who enjoy it for achieving more privacy that is not always guaranteed by other SNS. However, further studies should confirm our hypothesis and our findings in order to build targeted preventive interventions to specifically address SnapChat users at-risk for NSSI conducts. Conversely, according to our findings, being a male, having a higher educational level and an older age at the first time looking for NSSI-related contents on SNS seemed to act as possible protective factors for the development of NSSIs, as already documented in previous literature (28, 33).

Despite the abovementioned promising findings and the valuable insights provided into the association between social media use and engagement with NSSI content on SNS among young adults, it is necessary to be prudent before considering these findings generalizable to the clinical and other nonclinical samples, by carefully addressing and discussing all potential limitations. Firstly, while PSMU and FoMO appeared to be potentially associated with a higher risk to develop NSSI conducts, the cross-sectional design of the study did not allow to draw definitive conclusions regarding their causal relationship. Hence, further studies should carefully evaluate whether PSMU and/or FoMO are consequent or risky causal factors for the development of NSSI-related content behaviors and/or NSSI conducts. In fact, while individuals with higher levels of PSMU and FoMO could be more likely prone to seek out NSSI-related contents on SNS, it is equally plausible that those already predisposed to NSSI behaviors are drawn to such content regardless of their social media habits. Therefore, the directionality of the relationship between social media use and NSSI engagement remains a topic for further investigation, by implementing longitudinal studies. Secondly, the use of self-report assessment tools could potentially determine a response bias and inaccuracies in data collection, despite the anonymous data collection on the web could in turns predispose a higher participants’ openness in providing relevant information on a particularly sensible topic such as NSSI. On the other hand, participants could have underreported or overreported their social media use and engagement with NSSI content due to social desirability bias or memory recall errors. Therefore, our findings should be adequately replicated also in clinical samples and by an in-person recruitment strategy, by also integrating the assessment tools with clinician-guided semi-structured interviews. Thirdly, our sample is represented by young adults (aged 18–24) coming from a representative sample of Italian young adults, mainly represented by females. Therefore, one could argue that our findings should be properly replicated by comparing all age groups with each other and by including a sex-balanced sample, in order to explore whether these findings are only age and/or sex-specific within the Italian general population. Fourthly, other variables have not been investigated, such as the age of the first use of smartphone, Internet, the family context and the use of these tools within the family members, the attachment style and other personality features of participants, cultural differences in social media use, attitudes towards mental health and the norms surrounding NSSI behaviors, as well as to screen the sample according to a previous and/or current history for a personal and/or family psychiatric disorder. Furthermore, our study lacks to fully investigate the role of potential ‘protective factors’, such as a concomitant psychotherapy and/or psychological support, the family context, other personality variables such as the level of interpersonal sensitivity, resilience, and so forth. Further studies should also assess and consider not only risky but also potential protective factors in the onset and maintenance of SNS-driven NSSI conducts. Lastly, while the study identifies certain social media platforms, such as Snapchat, as significant predictors of NSSI behaviors, it should be considered that social media platforms are extremely dynamic and constantly evolving, hence, these findings should be furtherly implemented and replicated over the time based on the emergence of new SNS and functionalities. Finally, another potential bias could be represented by the lack of replies to the questionnaire item assessing previous contacts with a mental health professional, which was a not mandatory item to be filled out, which did not receive a sufficient number of replies to be considered in our analysis. Therefore, a further study should also consider this variable in order to stratify the sample accordingly.

Overall, our study confirmed the complex interplay between social media use, NSSI-related content search on SNS, and engagement with NSSI conduct among youngsters. These findings could help in addressing focused preventive and treatment strategies that necessarily require a multifaceted age-tailored and sex-based approach that encompasses both individual-level interventions and structural changes to mitigate the harmful effects of social media on more vulnerable people (particularly, transgenders people, non-binary subjects, females and pre-adolescents). Further research is needed to explore the longitudinal effects of social media use, PSMU and FoMO on NSSI-related mental health outcomes and inform evidence-based interventions aimed at promoting positive online behaviors and reducing the occurrence of NSSI-related contents on SNS as well as the propensity to actively look for NSSI-related con rTMS on SNS and subsequently to increase the likelihood to drive more vulnerable youngsters towards the development of NSSI behaviors.

Future research should be implemented in order to better explore all various avenues (risky and protective factors) derived by our findings as discussed above. These include also investigating cultural influences on the use of SNS platforms both for looking for NSSI-related contents for curiosity and for learning how to practice them in real life. In this regard, another research direction should clearly provide a multicentric and multi-ethnic/cultural stratification sample able to stratify the risk in being engaged in NSSI-related contents and the consequent risk to determine NSSI conducts, considering also the country-based differences. Particularly, it would be interesting also to evaluate the ‘migration effect’ as well as the variable to belong to the next generation following a migratory flux in mediating the relationship between the quest for NSSI-related contents and the consequent risk to use these contents to incentivize NSSI conducts and/or suicidality. Moreover, another research direction should also include to clearly examine whether PSMU could act as predictor or rather a consequence of the behavior to actively look for NSSI-related contents in SNS, in order to better understand how to properly address preventive measures. Another research direction should clearly investigate the role of concomitant psychological and/or psychopathological conditions in predisposing youngsters towards the propensity to use SNS as a mean to cope with these states and consequently use SNS to look for NSSI-related contents with the aim to find a mean to manage distress and/or alleviate an anxiety and/or depressive condition. Furthermore, another research direction should carefully consider the role of sex orientation in depending and investigating the role of SNS in privately managing concomitant stigma-related psychological conditions in this vulnerable population, in order to develop specific preventive and supporting interventions and policies to prevent suicide and self-injury among youngest but also considering the LGBTQI+ population.

Overall, our findings confirmed the importance and the urgent need to fully understand the complex interplay between social motivations, psychological factors, and online behavior in predicting engagement of youngsters with NSSI-related contents and RSMCs. While this study sheds light on the intricate relationship between social media use and engagement with NSSI-related contents on SNS among youngsters, it also underscores the need for ongoing exploration and collaborative efforts to address the unpredictable challenges posed by digital platforms. By fostering digital literacy, implementing robust SNS content moderation, and providing accessible psychological and psychiatric SNS-driven supporting services, institutions and clinicians could potentially strive towards creating safer online environments promoting mental well-being. However, as all currently are invested and shaped by the ever-evolving, fluid and dynamic virtual landscape and dynamics between social media and mental health, one could argue that there is always an urgent need to investigate the techno-bio-psychosocial model of mental health considering not only the detrimental effect of technology and SNS but also its potentiality in shaping the future of mental health, posing innovation and a digital collective action as means to shape a more compassionate digital future for all.

## Data availability statement

The datasets presented in this article are not readily available due to identification of sensible data. Requests to access the datasets should be directed to LO, l.orsolini@staff.univpm.it.

## Ethics statement

The studies involving humans were approved by Local Institutional Review Board of the Department of Experimental and Clinical Medicine/DIMSC, Polytechnic University of Marche, Ancona, Italy (protocol code ACPS-D-21-00347, 28th September, 2021). The studies were conducted in accordance with the local legislation and institutional requirements. The participants provided their written informed consent to participate in this study.

## Author contributions

LO: Conceptualization, Data curation, Formal Analysis, Investigation, Methodology, Supervision, Validation, Writing – original draft, Writing – review & editing. SR: Data curation, Formal Analysis, Investigation, Resources, Software, Writing – original draft. GL: Data curation, Formal Analysis, Methodology, Software, Writing – original draft. UV: Supervision, Validation, Visualization, Writing – review & editing.
